# Half a century of bioethics and philosophy of medicine: A topic‐modeling study

**DOI:** 10.1111/bioe.13087

**Published:** 2022-09-28

**Authors:** Piotr Bystranowski, Vilius Dranseika, Tomasz Żuradzki

**Affiliations:** ^1^ Interdisciplinary Centre for Ethics & Institute of Philosophy Jagiellonian University in Kraków 31‐044 Kraków Poland

**Keywords:** bioethics, distant reading, philosophy of medicine, topic modeling

## Abstract

Topic modeling—a text‐mining technique often used to uncover thematic structures in large collections of texts—has been increasingly frequently used in the context of the analysis of scholarly output. In this study, we construct a corpus of 19,488 texts published since 1971 in seven leading journals in the field of bioethics and philosophy of medicine, and we use a machine learning algorithm to identify almost 100 topics representing distinct themes of interest in the field. On the basis of intertopic correlations, we group the content‐based topics into eight clusters, thus providing a novel, fine‐grained intellectual map of bioethics and philosophy of medicine. Moreover, we conduct a number of diachronic analyses, examining how the “prominence” of different topics has changed across time. In this way, we are able to observe the distinct patterns in which bioethics and philosophy of medicine have evolved and changed their focus over the past half a century.

## 1 INTRODUCTION

One of the founding figures of bioethics noted that “Disciplines are not born; they grow slowly, gradually taking a shape distinct enough to merit a name.”^1^ In this paper, we investigate how the broad field of bioethics and philosophy of medicine has gradually taken shape over the last half a century, after having been consolidated as an institutionalized discipline in the 1970s.

We assume that the history of bioethics and philosophy of medicine has been characterized by the content published by the journals that the research community finds the most influential. The role of journals, because of their rapid production cycle and diversified content, may be more important for the developments in bioethics than monographs, which are essential for many other areas of the humanities or social sciences. First, the practical aims of the important part of this discipline mean that it aspires not only to understand and react quickly to scientific or technological developments, policy decisions, and societal changes but also to influence the legal regulations of biomedical research and clinical practice. Second, the interdisciplinary character of this field (or rather its role as a meeting place of disciplines) not only provides a discussion platform where humanistic insights and social sciences methods face the growing intricacy of biomedical technologies but also includes the voices of practitioners from outside of academia. Such diversity is clearly discernible in the publishing patterns of many leading journals, for example, the *Hastings Center Report*, the very first bioethics journal established by Dan Callahan in 1971.

The history of bioethics is tightly interwoven with that of its older sibling, the philosophy of medicine, which provides philosophical foundations for many bioethical debates. The inseparability of these two is often highlighted by scholars writing about the history of the intersection of medicine and the humanities. For example, Robert M. Veatch noticed “the gradual convergence of themes in philosophy of science and philosophy of medicine with more specific issues in medical and biomedical ethics.”^2^ It is also visible in the self‐declaration of *The Journal of Philosophy and Medicine*, the second oldest bioethics journal in the United States, which defines itself as “the flagship scholarly journal in bioethics and the philosophy of medicine.”

A standard manner in which practitioners of an academic discipline reflect upon the history and development of their own discipline is through “close reading” of selected texts, which is often mediated by their personal experience and academic interests. This approach is present in the classic books about the history of bioethics^3^ or important articles that try to identify “the hottest topics” during the development of the field.^4^ Here is a typical statement identifying trends in bioethics based on such an approach: “Over the course of the history of bioethics certain topics have moved in and out of fashion: in the 1970s it was euthanasia and abortion, in the 1980s genetics, in the 1990s stem cells and reproductive technologies, and in the 2000s, enhancement and data/tissue storage.”^5^ However, “close reading” as a way to detect very general trends in the literature is sometimes treated not only as not replicable and suffering from underdetermination by evidence (i.e., different interpretations may easily be drawn on the basis of the same material) but first of all as nontransparent and one that uses arbitrary sampling when working with large literatures.^6^

In contrast, the approach that we adopt in this article takes seriously the epistemological question of how one can justify the belief, for example, that the issue of “enhancement and data/tissue storage” dominated the debates of the 2000s. We use a “distant reading”^7^ approach based on topic modeling—a computational text‐mining technique aimed at discovering hidden thematic compositions in large text corpora. We believe that this technique provides a rigorous tool for understanding the structure of bioethics and philosophy of medicine (as well as their development over the last half a century) represented by the content published by seven leading journals, as identified by experts in the field.

The latent Dirichlet allocation (LDA) algorithm, which we use in this study, identifies “topics,” that is, sets of words that tend to be used together across documents in the corpus.^8^ Those “topics” are chiefly characterized by the relatively small sets of words most strongly associated with them, and, thus it is typically easy for the researcher to *interpret* them, that is, to associate “topics” with actual, discrete themes discussed in the analyzed collection of texts. For instance, if the model's output includes a topic characterized by the terms “gene,” “therapy,” “clone,” “disease,” and “germline,” we can reasonably interpret such a topic as being connected to the classic debate on germline modification and gene therapy.

A topic model is able to provide the exact proportions in which different topics discovered by the model contribute to each document in the corpus. This makes a number of interesting analyses easy to conduct: Which topics are the most prominent in the corpus? Which topics tend to occur together in the same documents? How does the average prominence of a given topic change for documents from different periods? These are the kinds of analyses that make topic modeling so useful in analyzing large bodies of scholarly texts.^9^

We assume that this method allows us to uncover the pattern of researchers’ interests and the evolution of such interests over time. Our aim is not to replace close reading, which is so typical for the humanities, but rather to present an instrument useful for researchers that may support human interpretive work “by providing evidence for interpretations in a manner that is not only much more scalable but also less subject to biases that derive from the interpreters’ preconceptions.”^10^ In other words, we are able to analyze the themes that “have moved in and out of fashion” in bioethics and philosophy of medicine more precisely and rigorously than with the help of standard “close reading” methods. Assuming that the prominence of different topics in our corpus is a proxy for the popularity of different bioethical themes among researchers, the method used in this paper may be helpful in interpreting the thematic structure of the entire field, the relations between different themes, and its diachronic changes. Moreover, as we observe that some topics are correlated, in the sense of being more frequently present together in the same texts and thus creating interconnected clusters of related topics, we end up drawing a novel, fine‐grained yet interesting map of bioethics and philosophy of medicine that readers should inspect in full on their own.^11^ Still, in Section 4, we comment in more detail on some of the observed patterns that we think are particularly unexpected or interesting. Before doing so, we present and analyze our main findings.

## 2 CORPUS AND METHODOLOGY

### 2.1 Journal selection

Following similar analyses conducted in other areas of philosophy,^12^ we aimed to fit a topic model on a corpus of full texts of all articles published in leading journals in the field of bioethics and philosophy of medicine.^13^ However, given the number of outlets publishing research in this field, any selection of target journals based on our own judgment would risk representing our personal preferences rather than the actual role played by the given journals. Hence, we chose instead to base the selection on more objective criteria.

To establish the list of the most representative journals, we invited experts in bioethics or philosophy of medicine to conduct a free listing task. A request to provide a list of five key journals in philosophy of medicine and/or bioethics was distributed via the Philos‐L mailing list (a large mailing list focused on philosophy‐related news) and—after the initial round of data collection—posted on the “Bioethics International” Facebook page and tweeted out by our department's profile. The following criteria were provided to specify what we mean by “key journal in philosophy of medicine and/or bioethics”:

a) The journal is focused on the general philosophy of medicine and/or general bioethics rather than a narrower and more specialized subfield.
b) The journal played an important role in shaping the field.
c) The journal publishes important work in the field.
d) The journal is recognized by the community as a key journal in the field.

**Figure 1:**
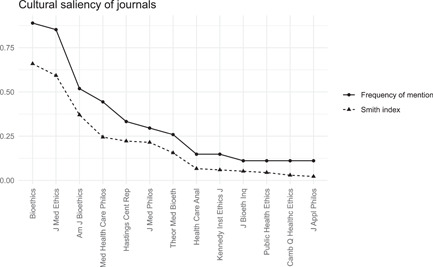
Frequency of mention and cultural saliency (Smith index) of key journals in philosophy of medicine and/or bioethics in our sample of experts (*N* = 27). Journals that were mentioned by fewer than three experts are not included in the graph.

We received responses from 27 individuals who indicated that they are “teachers and/or researchers in an academic institution.” To analyze the free‐listing data, we used FLARES.^14^ Every expert provided a list of five journals and this resulted in a list of 135 items. Twenty‐eight different journals were mentioned. Figure 1 presents the results of the free‐listing analysis. The frequency of mentions (solid line) indicates the proportion of experts who mentioned a given journal, with the highest numbers for *Bioethics* (89%) and the *Journal of Medical Ethics* (85%). The Smith index (dotted line) is a measure of cultural saliency that combines frequency of mention and rank of citation of items on the lists, that is, how early in the lists a journal tends to be mentioned.^15^

On the basis of the free‐listing analysis, we included the following seven journals (frequency of mention in brackets): *Bioethics* (89%); the *Journal of Medical Ethics* (JME, 85%); the *American Journal of Bioethics* (AJOB, 52%); *Medicine, Health Care and Philosophy* (MHCP, 44%); *Hastings Center Report* (HCR, 33%); the *Journal of Medicine and Philosophy* (JMP, 30%); and *Theoretical Medicine and Bioethics* (TMB, 26%).^16^ We decided to choose seven journals because of a clear drop in the frequency of mentions and the Smith index between the seventh journal and the eighth journal.^17^

### 2.2 Corpus acquisition and characteristics

Having identified the target journals, we built a complete corpus of texts published in all seven journals. We included regular‐length articles but also many types of shorter pieces because of their relative importance in the field of bioethics: open‐peer commentaries, replies, letters, book reviews, and so forth. We believe that these types of publications are particularly important in bioethics as it is a practically oriented discipline. However, we excluded types of documents that would typically lack “substantive” content: tables of contents, issue introductions, corrigenda, lists of referees, book notes, calls for papers, obituaries, and so forth.^18^ We also excluded extremely short documents (below 3000 characters) independently of their content. Only the main text of a document was to be included in the corpus. This meant that we aimed to exclude other elements of a document: title, abstract, authors list, reference list, footnotes, endnotes, acknowledgments, and so forth.

**Figure 2:**
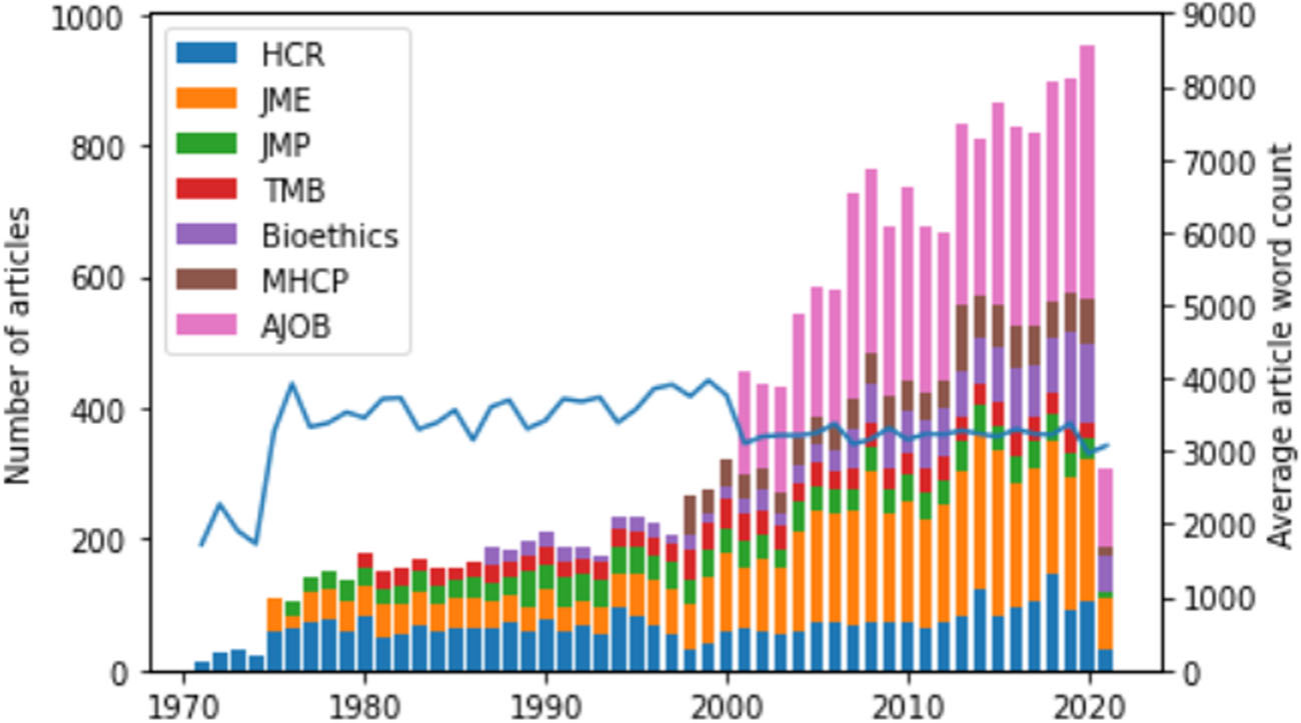
Number of articles per journal and the average article word count (the last column denotes articles published in the first 3 months of 2021).

The resulting corpus consisted of 19,448 documents, with 64,326,072 tokens (words) distributed across them. The average length of the main text of a document in the corpus was 3308 words, following the relative prevalence of shorter texts. The vast majority of documents came from three journals: AJOB (27%), JME (26%), and HCR (18%), with the four remaining journals contributing between 6% and 8% each. The time distribution of documents was also rather skewed, with few texts published in the 1970s (4%), the 1980s (9%), or the 1990s (11%) and the vast majority published in the 2000s (28%) and from 2010 onwards (41%). The number of articles in each journal and the average article word count are plotted in Figure 2.

We built the corpus in April 2021 and fitted the topic model on all eligible texts published as of that date.

### 2.3 Corpus cleaning and preprocessing

Whenever possible, we scrapped text from the HTML version of a document from the publisher's website. For documents where it was not possible, we downloaded a PDF version of the article and scrapped the text using GROBID software.^19^ We used regular expressions to remove inline references, as well as any residual footnotes, lists of references, copyright notes, URL addresses, and so forth, from the corpus. We lower‐cased tokens and removed punctuation and numerals. We removed stopwords^20^ by using the list of 179 English‐language stopwords from the Python *NLTK* library.^21^ We used the function *Phrases()* from the Python *GenSim* library^22^ to detect common multiword expressions, with the PMI‐like scoring^23^ of 100 or above, and transformed them into bigrams or trigrams (e.g., as words *male* and *circumcision* tended to occur next to each other, each such occurrence was transformed into a single bigram: *male_circumcision*). Then, we used the spaCy tagger^24^ to identify the part of speech of every token to discard all tokens that were not nouns, verbs, adjectives, adverbs, or proper nouns (hence, we removed determiners, prepositions, pronouns, etc.). Finally, we conducted lemmatization^25^ using the Python library spaCy.^26^ The resulting dictionary consisted of 173,489 terms.

However, the vast majority of those appeared in only a couple of documents, which meant that they would not be very useful in fitting a topic model (or they would even be a source of noise). For this reason, we chose to keep only those token types that appeared in at least 25 documents from the corpus and in no more than 50% of all the documents, which allowed us to substantially reduce the size of the dictionary to 20,005 terms. The distribution of those terms across all 19,448 documents (i.e., the *document‐term matrix*) was the main input to the topic modeling algorithm.

### 2.4 Topic modeling

To fit the topic model, we used the standard LDA algorithm^27^ with Gibbs sampling, as implemented in the Python library *lda*.^28^ LDA is among the oldest and simplest topic modeling algorithms, but it remains the most established and widely used tool in this context.^29^ LDA assumes that the analyzed corpus consists of a set of topics, where each topic is a probability distribution over the entire vocabulary used in the corpus. Terms (words and collocations) assigned a high probability within a single topic tend to co‐occur in corpus documents more frequently than would happen by chance. Furthermore, LDA associates each document in the corpus with a distribution over topics, thus showing the proportions of the topical composition of a given document. Crucially, the algorithm searches for distributions that would facilitate two (conflicting) goals: first, that each topic assigns a high probability to just a few terms (reflecting the intuition that distinct themes are characterized by a small set of key words) and, second, that each document assigns high probability to just a few topics (reflecting the intuition that each document engages with a small number of main themes).

LDA is an “unsupervised” algorithm, which means that resulting topics are not in any way guided by researchers’ expectations but, instead, are “discovered” by the algorithm itself on the sole basis of the patterns of co‐occurrence of terms across documents. Researchers, however, still have some control over the operation of the algorithm, as they have to decide on three hyper‐parameters: *alpha* (which controls prior topic probability distributions over documents), *beta* (which controls prior term probability distributions over topics),^30^ and, most importantly, *K* (the hypothesized number of topics in the corpus). As for alpha and beta, following some fine‐tuning, we chose the rather standard values of 0.01 and 0.31. As for *K*, we followed a standard practice of manually comparing the output of models with different numbers of topics and picking the one that, according to our judgment, appeared optimal. Typically, the optimal number of topics is neither too low (as this would result in large, heterogeneous topics that are hard to interpret) nor too high (as this would lead to topics that are overlapping and too numerous to make the entire model comprehensible for humans). In the present context, we fitted 10 models (for *K* = 30, 40, 50, 60, 70, 80, 90, 100, 110, 120) and decided that the model with 100 topics looked the most promising, with almost all of the resulting topics corresponding to what we find to be distinct themes that are present in the literature.^31^

### 2.5 Interpreting and clustering the topics

A topic resulting from an LDA algorithm is nothing more than a probability distribution over terms (words and collocations), so it does not have any determined *meaning*—it has yet to be interpreted by researchers. The interpretative task is made easier by the fact that the LDA algorithm guarantees that the probability mass of each such distribution is focused on a relatively small number of highly probable words, so that it is just a small number of such words that characterize a given topic and should suffice for its interpretation. For example, in the context of topic 87, a glimpse at the set of five most probable terms (“virtue,” “action,” “character,” “pellegrino,” “aristotle”) was sufficient for us to interpret that topic as referring to virtue ethics.^32^ Such interpretations can be guided or corroborated further by examining the documents most strongly associated with a given topic, as just a few documents for which a given topic is the most probable should characterize this topic quite well.^33^

In almost every topic model, however, a fraction of the topics resist such an interpretation as they represent stylistic peculiarities or other possibly spurious patterns present across documents. We found three such topics in our model (3; 9; 66) and discarded them from further analysis.^34^ Some further topics seemed either to refer to specific types of journal texts (60: *Clinical stories*; 71: *Reviews*) or to denote specific methodological approaches used in different contexts (2: *Concepts*; 12: *Qualitative*; 24: *Quantitative*; 49: *Moral philosophy*); following an earlier study,^35^ we call those “framing topics” and treat them separately in most of the analyses to follow.^36^ The list of all interpreted topics with a longer descriptive title and the corresponding 10 most likely terms for each topic can be found in Appendix A.^37^

This still left us with 91 topics that we interpreted as denoting distinct areas of research present in the target journals. Such a wide class of topics is not particularly manageable; hence, we aimed to reduce its dimensionality by following the procedure used earlier by Malaterre and colleagues.^38^ On the basis of document‐topic probability distributions, we calculated pairwise intertopic correlations.^39^ The resulting correlation matrix was the basis for constructing a graph in which nodes represent topics and edges represent intertopic Pearson's correlation coefficients above 0.05.^40^ We ran a series of modularity analyses—that is, we used the community detection method proposed by Blondel and colleagues, as implemented by Bastian and colleagues^41^—until we found a solution whose eight clusters seemed easily interpretable as distinct greater areas of research in bioethics and philosophy of medicine.

As a result, we have the following eight clusters of topics: BEGINNING OF LIFE; END OF LIFE; INSTITUTIONS; PATIENTS AND RESEARCH PARTICIPANTS; PHILOSOPHY OF MEDICINE; PHYSICIAN AND RESEARCHER; EMERGING TOPICS; and THEORETICAL BIOETHICS.

**Figure 3:**
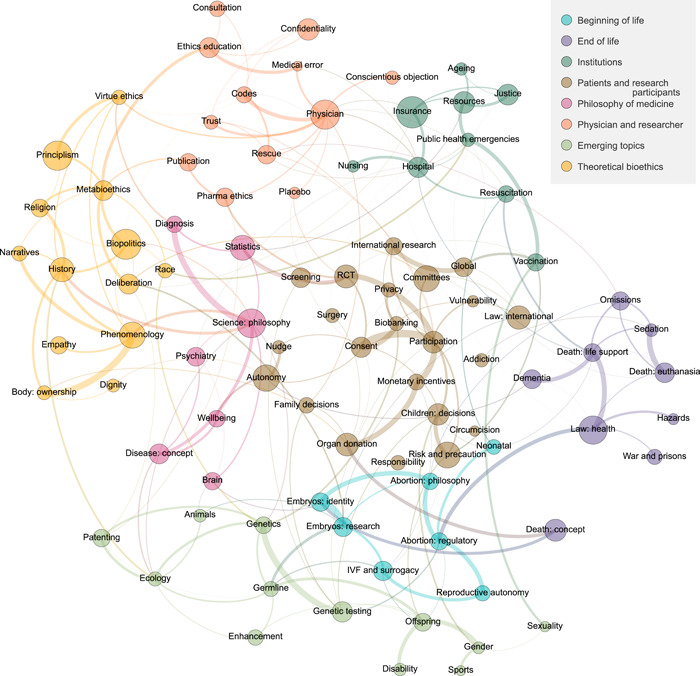
Ninety‐one content‐based topics grouped into eight clusters. Node size reflects a topic's prominence in the corpus, and edge size reflects Pearson's correlation coefficient for a given pair of topics (only correlation coefficients above 0.05 are included in the graph). Gephi's Multigravity ForceAtlas 2 was used for layout rendering.

On the basis of our expert judgment, we chose to manually correct the output of the clustering algorithm in cases where the assignment of a given topic to a given cluster seemed to be based on correlations that we interpreted as accidental. We therefore proceeded—mostly guided by correlations with other topics—to move three topics originally in the cluster PHILOSOPHY OF MEDICINE (*Dignity* to THEORETICAL BIOETHICS; *Autonomy* and *Nudge* to PATIENTS AND RESEARCH PARTICIPANTS), five topics from BEGINNING OF LIFE (*Death: concept* to END OF LIFE; *Organ donation*, *Monetary incentives*, *Surgery*, and *Family decisions* to PATIENTS AND RESEARCH PARTICIPANTS), one topic from INSTITUTIONS (*Sexuality* to EMERGING TOPICS), and one topic from END OF LIFE (*Addiction* to PATIENTS AND RESEARCH PARTICIPANTS). The resulting classification is visualized in Figure 3, and the assignment of individual topics to clusters can also be found in Appendix A.

## 3 ANALYSES

### 3.1 Topic prominence

Topics differed widely in their overall prominence,^42^ ranging from 0.28% (*Sports*) to 1.86% (*Insurance*). The most prominent topics in the present corpus were *Insurance* (1.86%), *Law: health* (1.85%), *Physician* (1.78%), *Committees* (1.78%), *Biopolitics* (1.47%), *Principlism* (1.47%), *Risk and precaution* (1.43%), *RCT* (1.37%), *Autonomy* (1.37%), and *Confidentiality* (1.35%). Joint prominence of all content‐based topics as a group is 77.41%. The most prominent topics within each cluster are the following: BEGINNING OF LIFE: *Embryos: research*, 0.91%, *IVF and surrogacy*, 0.89%, *Abortion: regulatory*, 0.83%; END OF LIFE: *Law: health*, 1.85%, *Death: euthanasia*, 1.03%, *Death: concept*, 1.03%; INSTITUTIONS: *Insurance*, 1.86%, *Hospital*, 1.23%, *Resources*, 1.11%; PATIENTS AND RESEARCH PARTICIPANTS: *Committees*, 1.78%; *Risk and precaution*, 1.43%, *RCT*, 1.37%; PHILOSOPHY OF MEDICINE: *Statistics*, 1.22%, *Science: philosophy*, 1.20%, *Brain*, 0.82%; PHYSICIAN AND RESEARCHER: *Physician*, 1.78%, *Confidentiality*, 1.35%, *Ethics education*, 1.25%; EMERGING TOPICS: *Genetic testing*, 1.12%, *Patenting*, 0.87%, *Offspring*, 0.78%; THEORETICAL BIOETHICS: *Biopolitics*, 1.47%, *Principlism*, 1.47%, *Metabioethics*, 1.25%.

### 3.2 Prominence of topic clusters

We also calculated the joint prominence of topic clusters by adding together the prominence of all individual topics that constitute a given cluster. Clusters range in prominence from 5.25% to 20.37%: PATIENTS AND RESEARCH PARTICIPANTS (20.37%), THEORETICAL BIOETHICS (11.44%), PHYSICIAN AND RESEARCHER (10.67%), INSTITUTIONS (8.50%), END OF LIFE (8.17%), EMERGING TOPICS (7.06%), PHILOSOPHY OF MEDICINE (5.96%), and BEGINNING OF LIFE (5.25%).

### 3.3 Prominence of framing topics

Topic prominence for framing topics ranged from 1.29% to 3.10%: *Moral philosophy* (3.10%), *Concepts* (2.55%), *Clinical stories* (2.17%), *Reviews* (1.63%), *Qualitative* (1.30%), and *Quantitative* (1.29%). Overall, joint prominence of framing topics is 12.04%.

### 3.4 Intertopic correlations

A table of the Pearson's correlations between topics (measuring the tendency of a pair of topics to co‐occur in the same documents) is provided as a supplement.^43^ Here is the list of the 10 most strongly correlated pairs of topics: *Consent*—*Participation* (*r* = 0.19), *Phenomenology*—*Body ownership* (0.17), *Embryos: identity*—*Embryos: research* (0.16), *Monetary incentives*—*Participation* (0.16), *Genetics—Genetic testing* (0.16), *Science: philosophy—Diagnosis* (0.15), *Death: euthanasia—Sedation* (0.15), *Participation—RCT* (0.15), *Biobanking—Consent* (0.14), *Monetary incentives—Organ donation* (0.14), and *Disease: concept—Concepts* (0.14). Because clusters were based on intertopic correlations, it is unsurprising that all these pairs connect topics within the same cluster (except *Disease: concept—Concepts*, which involves a framing topic *Concepts*). Overall, positive correlations were more pronounced than negative ones (the strongest negative correlation was *Moral philosophy*—*Clinical stories*, *r* = −0.10).

### 3.5 Diachronic analysis of topic prominence

We conducted our diachronic analyses for the period from 1976 (the year in which JMP began publication, after HCR and JME, which were already in production) to 2020 (the last year for which we had a complete set of articles published by all seven journals). To focus on long‐term trends and avoid noise caused by factors such as the publication of special issues, we divided that 45‐year period into nine 5‐year periods and calculated each topic's *prominence in a respective period* (i.e., the mean probability with which an article published in a given 5‐year period expressed a given topic). Figure 4 shows diachronic plots of topic prominence for each of the 91 content‐based and 6 framing topics, grouped by clusters and, within each cluster, ordered by their overall prominence in the corpus. The area under the curve can be used to visually compare the overall prominence of different topics. As can be seen from the plots, the chronological development of topic prominence shows various patterns, from a gradual increase in prominence through relative uniformity over the years to a gradual increase. Some topics are suggestive of more complex chronological patterns, involving one or more peaks in prominence. We further explore these patterns in Sections 3.5.1, 3.5.2–3.5.3.

Figure 4The mean prevalence of topics across 5‐year periods from 1976 to 2020. Topics are grouped by clusters and, within each cluster, ordered by their overall prominence in the corpus.

**Figure:**
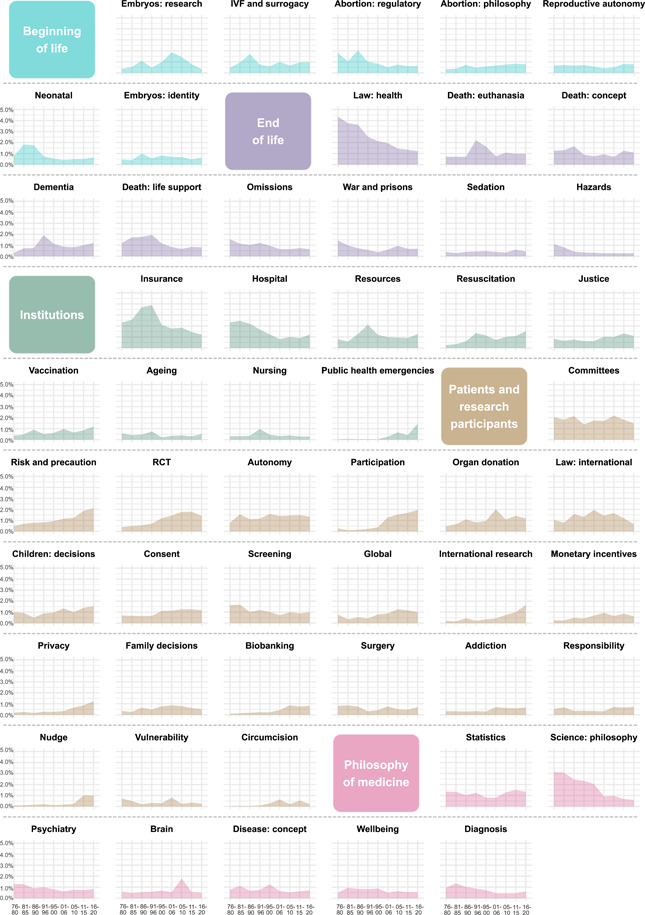

**Figure:**
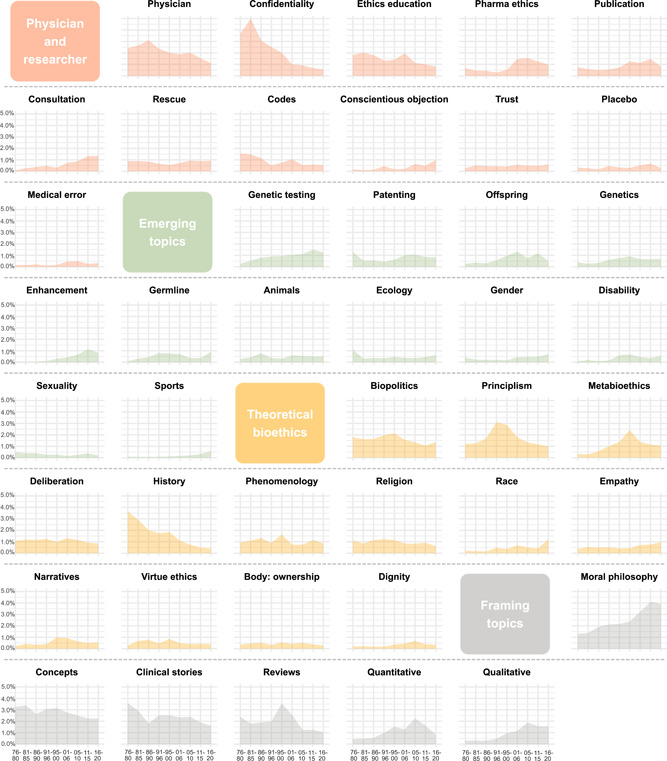

**Figure 5:**
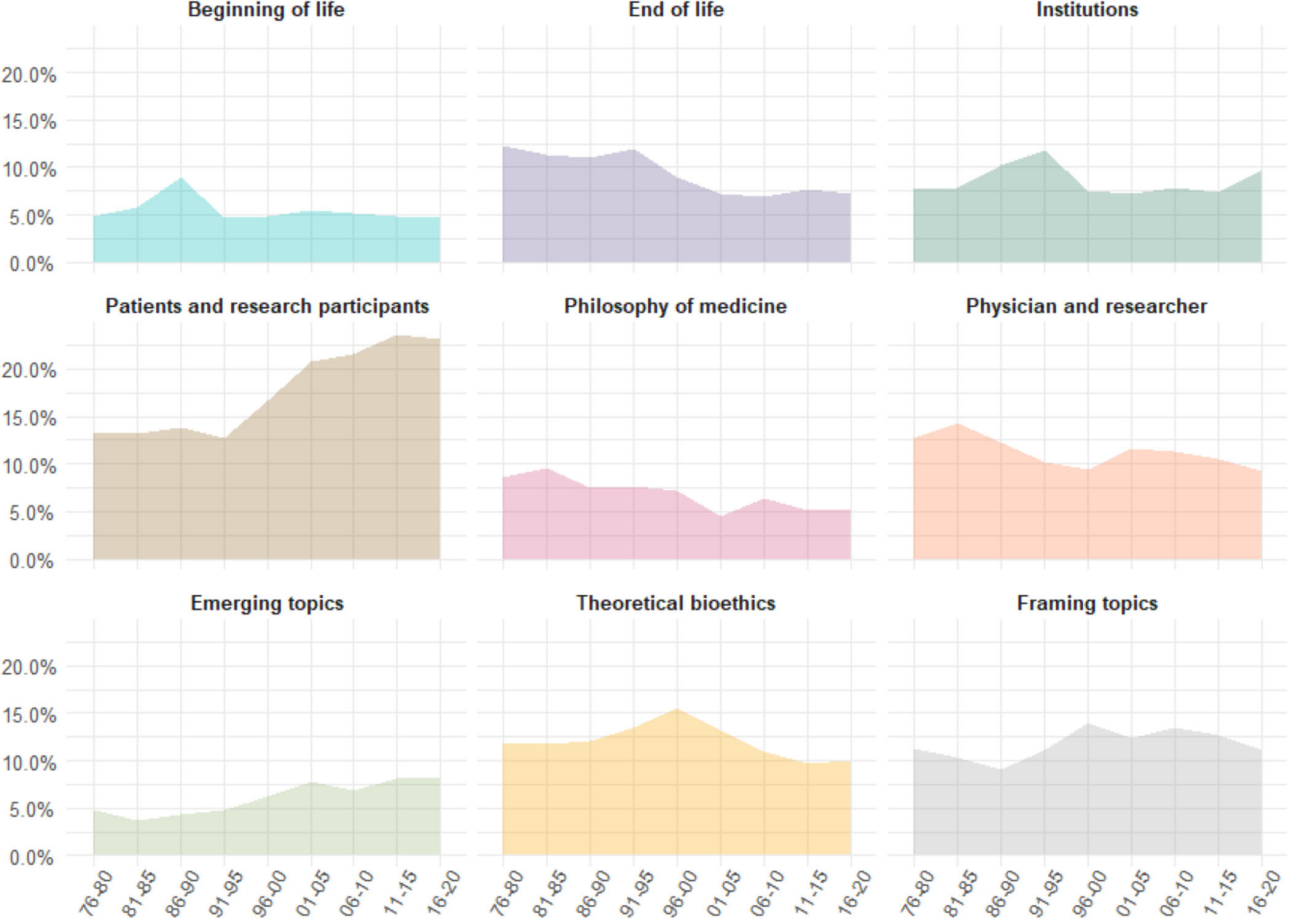
The mean prevalence of topic clusters (and framing topics) across 5‐year periods from 1976 to 2020

Figure 5 plots the mean prevalence of each topic cluster across 5‐year periods from 1976 to 2020. It also includes a separate plot for joint prominence of the six framing topics. Some topic clusters show a relatively clear growing or contracting pattern. For instance, we can compare the mean prominence of the cluster in the first two periods (1976–1985) to the mean prominence in the last two periods (2011–2020). EMERGING TOPICS and PATIENTS AND RESEARCH PARTICIPANTS in general seem to be gaining in prominence over time, with the mean prominence in the last two periods being, respectively, 194.13% and 177.13% of that in the first two periods. The most pronounced relative decline in prominence is that of PHILOSOPHY OF MEDICINE, followed by END OF LIFE, which have contracted by more than a third (to 56.10% and 63.36%, respectively).

#### 3.5.1 Largest overall increases and decreases

We looked at which topics demonstrated the largest increase and decrease in prominence overall. To do this, we compared the mean prominence of the topic in the first two periods (1976–1985) to its mean prominence in the last two periods (2011–2020). The 10 largest increases and decreases are presented in Table 1.^44^

**Table 1:** The biggest overall increases and decreases in topic prominence

Topic		Mean_1976–1985_ (%)	Mean_2011–2020_ (%)	Change (mean_2011–2020_/mean_1976–1985_) (%)
**Largest increases**				
*Enhancement*		0.03	0.97	3641.26
*Public health emergencies*		0.04	0.96	2490.50
*Circumcision*		0.04	0.37	1020.05
*Nudge*		0.10	1.00	990.73
*Participation*		0.19	1.83	986.98
*International research*		0.18	1.34	758.79
*Consultation*		0.18	1.33	730.69
*Biobanking*		0.12	0.80	678.90
*Conscientious objection*		0.13	0.75	581.33
*Sports*		0.08	0.46	577.57
**Largest decreases**				
*Confidentiality*		4.37	0.62	14.10
*History*		3.26	0.46	14.11
*Science: philosophy*		3.11	0.63	20.34
*Hazards*		0.94	0.28	29.17
*Law: health*		4.04	1.27	31.50
*Codes*		1.51	0.56	36.78
*Neonatal*		1.33	0.57	43.36
*Abortion: regulatory*		1.40	0.61	43.86
*Hospital*		2.39	1.05	43.96
*Diagnosis*		1.15	0.53	46.25

*Note*: Overall increase/decrease is defined as the mean prominence of a topic in the last two periods in our data set (2011–2020) divided by the mean prominence of the topic in the first two periods (1976–1985).

#### 3.5.2 Peaks

To identify and analyze any sudden increases in the prominence of topics—suggesting that a given topic had suddenly become notably more prominent—we developed a method of detecting peaks of topic prominence. The prominence of each topic in a given period was divided by the mean prominence of this topic for the two preceding periods (where a “period” is our 5‐year bin). We also applied a threshold of 1% prominence in the target period to focus on the most notable increases. Given our definition of a peak and the fact that our diachronic analysis starts in 1976, the first period for which we could identify peaks is 1986–1990. Table 2 provides a list of the twenty most substantial peaks, each showing at least a 2.25‐fold increase in the prominence of a topic.

**Table 2:** Highest increases of topic prominence

			Corpus			
			Full		Abridged	
Topic		Period	Increase: %	Prominence: %	Increase: %	Prominence: %
*Nudge*		2011–2015	409.31	1.15	316.08	0.73
***Embryos: research***		2001–2005	332.30	2.17	370.00	2.12
***Pharma ethics***		2001–2005	326.37	1.56	293.26	1.04
***Death: euthanasia***		1991–1995	323.06	2.31	407.01	2.53
***Participation***		2001–2005	320.45	1.47	405.46	1.37
***Public health emergencies***		2016–2020	316.92	1.68	286.00	1.26
***Germline***		2016–2020	312.57	1.05	359.64	1.19
*Offspring*		1996–2000	302.17	1.41	183.03	1.07
*Brain*		2006–2010	278.39	1.68	162.11	1.06
***Embryos: research***		1986–1990	265.31	1.18	280.40	1.30
***Dementia***		1986–1990	256.04	1.28	225.17	1.09
*Metabioethics*		1991–1995	254.06	1.29	198.64	0.81
*Consultation*		2011–2015	244.51	1.66	129.75	0.75
*Embryos: identity*		1986–1990	235.91	1.04	178.29	1.06
***Quantitative***		1991–1995	232.73	1.20	234.81	1.34
***Resources***		1991–1995	232.38	2.37	230.11	2.73
***IVF and surrogacy***		1986–1990	232.15	1.72	252.24	1.64
***Resuscitation***		1991–1995	229.18	1.35	264.63	1.14
*Race*		2016–2020	227.54	1.18	170.54	0.70
*Narratives*		1996–2000	225.07	1.15	194.51	1.02

*Note*: Increase is defined as the prominence of a topic in a given 5‐year period divided by the mean prominence of the topic for the two preceding periods. A threshold of 1% prominence in a target period is applied. Topics enhanced in bold made it into the top 20 in both full and abridged (texts that contain more than 2300 words) corpora.

Because our data set includes bundled texts, most prominently open peer commentaries appended to AJOB's target articles, we wanted to check whether peaks in the full corpus represent robust trends in the discussion rather than artifacts of multiple counting of such bundled short texts. We decided to conduct the same kind of peak analysis on an abridged corpus that contains only relatively long texts (at least 2300 words long).^45^ Twelve topics (60%) made it into both top‐20 lists (enhanced in bold in Table 2). The remaining eight topics still show a pattern of growth in the abridged corpus (the level of increase in prominence is in the range of a 1.3–3.2‐fold increase over the average of the previous two periods), but several of them no longer clear the 1% prominence threshold, with *Nudge* 2011–2015 and *Race* 2016–2020 being the least prominent (0.70%).

#### 3.5.3 Recent trends

The most recent trends are not always clearly visible in the above graphs based on 5‐year periods, so we decided to check for the most recent trends in a more fine‐grained manner. To identify any topics that are currently enjoying an increase in prominence within the corpus (Table 3), we calculated the average probability for each topic for the last two complete years in our data set (2019–2020), and we divided those values by analogical averages for the preceding 10‐year period (2009–2018).^46^

**Table 3:** Highest recent increases of topic prominence

Topic	Mean_2009–2018_ (%)	Mean_2019–2020_ (%)	Change (mean_2019–2020_/mean_2009–2018_: %)
*Public health emergencies*	0.43	2.85	664.99
*Germline*	0.42	1.28	304.18
*Privacy*	0.82	1.68	205.66
*Race*	0.67	1.25	187.34
*Disability*	0.41	0.73	178.30
*Vaccination*	0.82	1.41	171.46
*Gender*	0.55	0.90	162.98
*International research*	1.15	1.84	160.94
*Dementia*	0.96	1.53	158.93
*Abortion: philosophy*	0.70	1.07	153.69

*Note*: Recent increase is defined as the mean prominence of a topic in the last two complete years in our data set (2019–2020) divided by the mean prominence of the topic for the preceding 10‐year period (2009–2018).

## 4 GENERAL DISCUSSION

### 4.1 Contextualism and a document‐grounded approach

Before we discuss how one can use our data to interpret the topical structure and diachronic trends in bioethics and philosophy of medicine, we briefly discuss our main theoretical assumptions.

First, the method that we use assumes a contextual approach to meaning because co‐occurrences of terms in the same documents are crucial in the assignment of terms to topics. Moreover, each term is assigned with positive probability to many different topics, which may be interpreted as an ability of the method to capture polysemy or distinguish different uses of the same term on the basis of the context.^47^ The same is true about documents—their assignment to different topics may be interpreted as an ability of the model to capture the fact that documents are multithematic. This feature may be explained by means of the example of two topics that, respectively, we have termed (1) *Abortion: regulatory* (top‐10 terms: “abortion,” “fetus,” “woman,” “pregnancy,” “fetal,” “mother,” “birth,” “child,” “pregnant,” “prenatal”) and (2) *Abortion: philosophy* (“kill,” “status,” “future,” “abortion,” “fetus,” “wrong,” “personhood,” “morally,” “property,” “being”). These topics reveal somewhat different contexts in which the term “abortion” is placed.

The first use is more regulatory‐oriented, that is, connected with an institutional perspective; the second one is more theory‐oriented, that is, connected with a philosophical perspective on the moral (im)permissibility of abortion. This is clearly discernible if we compare the first uses of the word “abortion” in two papers that are most representative for the two respective topics. In the case of *Abortion: regulatory*, a relevant passage clearly refers to “abortion” in its institutional sense: “A growing number of states have banned abortion after twenty weeks on the grounds that ….”^48^ In contrast, in the case of *Abortion: philosophy*, a relevant passage refers to a more abstract sense of “abortion”: “a non‐religious argument against abortion based on what he claims is a morally relevant similarity between killing adult human beings and killing fetuses.”^49^ Therefore, the fact that we labeled multiple topics with the same main title but different subtitles may be illuminating for further interpretations (see also: *Death: life support*; *Death: concept*; *Death: euthanasia*).

Second, the natural consequence of our contextualism is a document‐grounded approach. Our preferred way of interpreting the topics, patterns, trends, and peaks in the corpus involves looking at documents themselves at every stage of the decision process that requires human interpretation. Let us again refer to the example with the two “abortion” topics. Comparing the top‐10 papers characteristic for these two topics is revealing because they provide evidence in favor of our interpretations of these two topics: In the first case, most documents refer to women's rights, *Roe v. Wade*, prenatal diagnosis, or the legal liability of physicians. In the second, most of the top papers discuss secular arguments against abortion, such as the “future like ours” argument proposed by Marquis and the substance view that concludes that a human fetus has the same intrinsic value as a typical adult human being.

### 4.2 The cluster structure of the field

What can researchers specializing in this field learn from a “distant‐reading” of this large corpus?

First, this method provides a data‐driven thematic partition of the field. Any attempt to do so on the basis of close‐reading the texts is much more susceptible to the biases of individual scholars—both because individual researchers cannot realistically read such vast collections of texts and because they cannot be expected to abstain from their own subjective takes on the relative importance of various themes. Intertopic correlations can allow us to see which topics are relatively more closely related, and the clustering of topics based on intertopic correlations offers a useful, and in some regards novel, largely data‐driven, eight‐part division of the field (see Figure 2).

In interpreting the cluster structure, we did not find it possible to interpret all the divisions within a unified categorization scheme. Rather, it seemed more natural to interpret various divisions between clusters as tracking a mix of somewhat different kinds of distinctions. The BEGINNING OF LIFE and END OF LIFE clusters seemed to cover topics traditionally referred to as ethical issues in the beginning and end of life. Both clusters are characterized by their object of study, but they bring together a heterogeneous set of perspectives: In addition to applied questions, such as the permissibility of abortion or euthanasia, they also cover more theoretical questions in the metaphysics of persons and personal identity. Second, the three clusters PATIENTS AND RESEARCH PARTICIPANTS, PHYSICIAN AND RESEARCHER, and INSTITUTIONS seem to span both sides of the research ethics/clinical ethics distinction and suggest a division on a different basis. Roughly, there are ethical issues centered on an individual who encounters the healthcare system (PATIENTS AND RESEARCH PARTICIPANTS); ethical issues centered on the professional agents within the healthcare system (PHYSICIAN AND RESEARCHER); and ethical issues centered on the institutional setting in which interactions between these two types of agents occur (INSTITUTIONS). Third, the distinction between PHILOSOPHY OF MEDICINE and THEORETICAL BIOETHICS seems to capture the distinction between topics characteristic of the general philosophy of science as applied to medicine and topics that are more theory‐oriented, covering moral and political philosophy as well as more general phenomenological and narrative approaches.^50^ Finally, EMERGING TOPICS is a cluster that seems to be mostly unified by the novelty and felt urgency of certain challenges—be they technological or societal. It is the cluster that was characterized by the greatest relative increase in prominence over time.^51^

Second, the method allows us to adopt a birds‐eye view of the development of the field. Diachronic changes in the relative prominence of both topics and their clusters can be a welcome addition to the more traditional tools used by historians of philosophy. We can learn which topics and topic clusters have gained or lost in relative prominence over time. Locating local peaks in relative prominence can allow for a more focused search for the precise factors that have shaped these discussions. This is particularly interesting to the extent that bioethics is said to develop in reaction to sudden shocks. For this reason, focusing on the most rapid changes in the topical composition can sometimes offer greater insight than studying long‐term trends.

### 4.3 The most prominent topics and the strongest correlations

At the most general level, our analyses suggest which areas of research attracted researchers’ attention in the last half century of bioethics and philosophy of medicine. Perhaps the most striking result is that the three clusters concentrated on regulatory and institutional issues related to healthcare systems and medical research (PATIENTS AND RESEARCH PARTICIPANTS; PHYSICIAN AND RESEARCHER; and INSTITUTIONS) are jointly more prominent than the remaining five content‐based clusters combined. This illustrates how heavily the whole field is invested in issues of clinical and research ethics.

At a more detailed level, our analyses can also show which topics in bioethics and philosophy of medicine were the most popular in general over the last 45 years (*Insurance*; *Law: health*; *Physician; Committees*). The fact that *Insurance*, *Law: health*, and *Committees* are among the three biggest topics reflects research interests in themes typical for the U.S. healthcare system. All of the top‐10 papers characteristic for *Insurance* discuss Medicaid, U.S. health reforms at different levels (federal and state), the healthcare programs of American presidential candidates, rising healthcare costs in the United States, and so forth. Similarly, in the case of *Law: health*, all of the most characteristic papers refer to the U.S. Constitution or mention some U.S. Supreme Court rulings. In turn, among the top‐10 papers in *Committees*, only one concerns a non‐U.S. context, and the remaining nine discuss The Common Rule, that is, the federal regulations of research with human subjects or the U.S. regulations about IRB. Bioethics and philosophy of medicine started in the United States as a discipline, and our analysis confirms that the themes important from the U.S. perspective loom large in it.

As to the pairs of most strongly correlated topics, they can serve more as a sanity check for the model rather than a source of surprising insights. It is hardly surprising that the topic *Consent* most typically accompanies *Participation* and *Biobanking* or that the topic representing the metaphysical discussion on the beginning of life (*Embryos: identity*) is strongly associated with *Embryos: research*. Perhaps more interesting is the list of most *negatively* correlated pairs of topics, which turn out to be dominated by framing topics. Here, two patterns can be observed. First, some pairs of framing topics are negatively correlated, suggesting that such perspectives are used very rarely in tandem to analyze an issue in bioethics and philosophy of medicine (*Moral philosophy* appears to be rarely combined with *Quantitative* or *Clinical stories*, and the latter also tends to dissociate with *Concepts*). Second, some framing topics are negatively correlated with some content‐based topics, suggesting that the latter are unlikely to be analyzed from a given perspective (and so, e.g., *Moral philosophy* seems to be rarely used in the context of *Hospital*, *Biopolitics*, *Law: health*, or *Screening*).

### 4.4 Diachronic trends

In the following pages, we briefly discuss some of the potential ways in which diachronic trends can be interpreted in two dimensions: (1) overall and recent trends and (2) peaks.

#### 4.4.1 Overall and recent trends

Diachronic analyses suggest changing patterns in research interests and reveal which themes have won or lost the attention of researchers over time. On the one hand, if we zoom in and focus on the popularity of particular themes in the early days of institutionalized bioethics and philosophy of medicine (1976–1985) in comparison with the last 10 years (2011–2020), “the biggest winners” in terms of relative growth are themes represented by the topics we called *Enhancement* (mean prominence from 0.03% to 0.97%), *Public health emergencies* (from 0.04% to 0.96%), and *Circumcision* (from 0.04% to 0.37%), whereas the greatest losers are *History* (from 3.26% to 0.46%), *Confidentiality* (from 4.37% to 0.62%), and *Science: philosophy* (from 3.11% to 0.63%).^52^

The first two winners (in terms of relative growth) are easily interpreted. In particular, taking into account that *Enhancement* is correlated with *Germline* and *Genetics* (see Figure 3), one can observe a broader trend of interest in different ethical, regulatory, and theoretical questions about heritable genome editing. *Germline* is also among the top recent peaks, but it is also perfectly understandable if one takes into account the recent explosion of interest in the CRISPR/Cas9 method and the He Jiankui scandal. Both of these issues were discussed in an article by Cwik, which is the most characteristic for this trend.^53^

The second topic among “the biggest winners” (*Public health emergencies*) is obviously related to COVID‐19, but the recent pandemic is not enough to do justice to the full scale of its growth. Even after excluding the “pandemic” year 2020 from the analyses (i.e., comparing 1976–1985 with 2011–2019), this topic would still be in the third place, just below *Circumcision*. It suggests that discussions about healthcare emergencies were steadily growing even before the COVID‐19 outbreak, which may be related either to earlier epidemiological crises, such as the 2013–2016 Ebola outbreak, or, more generally, to a growing interest in the bioethical aspects of large‐scale catastrophes, such as natural disasters or terrorist attacks.

The third winner, *Circumcision*, is an interesting case because all top‐10 articles characteristic for this topic are about two main issues: either neonatal male circumcision or female genital alteration. However, five of them were published in the same issue of *AJOB* (3(2)) and revolve around the target article by Benatar and Benatar.^54^ The paper reacted to discussions that were up to date at the time, including the statement issued in 1999 by the American Academy of Pediatrics and other guidelines published by medical societies, that highlighted the alleged health benefits of male circumcision—a view that has come to be seen as increasingly controversial. It seems that the relative growth of popularity of this topic in our corpus after 2000 may stem from the increased interest of bioethicists in the ethical issues around neonatal male circumcision, a practice that in the early days of bioethics and philosophy of medicine was not even considered to be ethically troubling (in contrast with the practices of female genital mutilation).

In the case of the biggest losers, a possible explanation for the relative decline of the topic that we called *History* may be found in the most characteristic papers for this topic. All of them refer to some classical texts (Gilgamesh) or authors (Socrates, Galen, Stoics, Boethius, Hume, Camus). One might speculate that the fact that the field is becoming more mature provides an explanation: In the early days of bioethics and philosophy of science, authors needed to refer to the classics, whereas now the main discussions concern one another's papers.

The relative decline of *Confidentiality* may be instructive because it may be interpreted as a sign that bioethics and philosophy of medicine were closer to physicians’ professional ethics in their early days and much more focused on physician–patient relations than now (which is further corroborated by the relative decline of *Clinical stories* and *Codes*, topics with which *Confidentiality* is correlated and which are related to the professional role of physicians). In turn, the relative decline of *Science: philosophy* may reflect a growing separation between bioethics and philosophy of science (this is further corroborated by the relative decline of *Diagnosis* and *Disease: concept*, topics with which *Science: philosophy* is relatively strongly correlated and which are also thematically close to philosophy of science). It seems that most papers characteristic for this topic could also be published in philosophy of science journals. This may be a sign that mainstream bioethics and philosophy of medicine journals are increasingly leaning toward an understanding of their own field as one that is more practice‐oriented than theory‐based.

On the other hand, we can also adopt a bird‐eye perspective and search for “the biggest winners” in terms of trends larger than a particular topic. From this perspective, the main winners are a group of topics that we interpreted as the cluster EMERGING TOPICS (covering topics that we called *Enhancement*, *Sport*, and *Genetic testing*, among others) and those areas of bioethics that are centered around an individual who encounters the healthcare system (i.e., the cluster PATIENTS AND RESEARCH PARTICIPANTS), whereas “the biggest losers” are topics characteristic of general philosophy of science as applied to medicine and related to the end of life (i.e., the clusters that we named PHILOSOPHY OF MEDICINE and END OF LIFE).

Particularly interesting is the relative expansion of the cluster PATIENTS AND RESEARCH PARTICIPANTS as well as the relative decline of END OF LIFE: in 1976–1980, both have a similar share in the corpus (13.15% and 12.26%, respectively), whereas in the last period (2016–2020), the first cluster represents 23.19% of the corpus, while the second represents only 7.3%. The relative growth of the first cluster is much easier to explain than the relative decline of the second.

The cluster PATIENTS AND RESEARCH PARTICIPANTS consists of many topics that are steadily gaining in popularity (or at least have done in recent years); in particular, *Circumcision* (mean prominence from 0.04% to 0.37%), *Nudge* (from 0.15% to 0.99%), *Participation* (from 0.19% to 1.83%), *International research* (from 0.18% to 1.34%), and *Biobanking* (from 0.12% to 0.79%) were “the biggest winners” in terms of relative growth (in ascending order while comparing the mean prominence in 1976–1985 with that in 2011–2020; all five are in top 10 of topics with the overall highest increases in topic prominence). Five topics from this cluster were more popular in the early days of bioethics than they are now, most notably *Vulnerability* (from 0.61% to 0.31%), *Screening* (from 1.65% to 0.94%), and *Surgery* (from 0.84% to 0.58%). However, to explain the growth of the cluster, absolute increases of the topic prominence are more meaningful. As mentioned above, all three content‐based topics that experienced the highest growth in absolute terms (*Participation*, *Risk and precaution*, *International research*) belong to PATIENTS AND RESEARCH PARTICIPANTS.

The explanation of the *Nudge's* dynamic (as well as its peak in 2011–2015) seems to be rather straightforward. Namely, it seems to reflect the uptake of the book by Thaler and Sunstein,^55^ which was thoroughly discussed by bioethicists both in the theoretical contexts of well‐established bioethics concepts such as informed consent, autonomy, or paternalism and in relation to more practical questions regarding the permissibility of nudging at the population level or in clinical contexts, for example, for organ donations. Even though the majority of top papers (9 out of the top‐10) refer to this influential book, it is worth noting that the topic is not limited to nudging but extends to more general debates on rational choice. One paper that does not even mention the word “nudge” is a commentary in AJOB to the important paper by Nelson and colleagues on voluntary consent.^56^

More intriguing is the enormous growth (10 times in terms of relative prominence) of *Participation*. Because most papers characteristic for this topic discuss fairness in the selection of research participants, it may reflect the growing interest and changing awareness of the inclusion of minorities, children, and pregnant women in research, something that has been gradually attracting attention over the last 20 years. Moreover, this topic also had an important peak in 2001–2005 that, judging from the top‐30 papers characteristic for this peak, was stimulated by the problems revolving around paying for research participation. Moreover, this topic is noticeably correlated with many others that have also been growing in popularity, for example, *Consent*, *Monetary incentives*, *RCT*, *Children: decisions*, *Risk and precaution*, suggesting a broader trend of interest in many ethical and regulatory issues in research with human participants.

The reasons why the cluster END OF LIFE as a whole has relatively shrunk is more difficult to explain. The strongest relative decline is visible in the cases of *Hazards* (from 0.94% to 0.25%), *Law: health* (from 4.04% to 1.27%); *Omission* (from 1.33% to 0.69%), *War and prisons* (from 1.2% to 0.67%), and *Death: life support* (from 1.46% to 0.83%). However, taking into account absolute decreases, the relative decline of END OF LIFE is largely driven by *Law: health*, which shrunk by 2.77 percentage points (the summary effect of the other four fastest shrinking topics was smaller than that of *Law: health*). Its most characteristic terms (e.g., “law”, “rule”, “judge”, “supreme_court”) are typical of papers published in the early days of bioethics, in particular in HCR, and now such issues may have moved to more legally oriented journals. However, the relative decline of the HCR's share in the corpus (in particular, after the launch of AJOB and the growth of JME in the 2000s) does not fully explain the decrease of this particular topic.

The relative decline of *Death: life support* is also surprising, particularly if one takes into account the fact that a similar topic, that is, *Death: euthanasia* (from 0.71% to 0.98%) is rather stable (with one important peak that we will discuss below). Anyway, this trend may be a sign that the popularity of one particular theme in discussions about aid in dying may be decreasing: Artificial nutrition and hydration were discussed in almost all top‐10 papers in the topic *Death: life support.* It is also worth noticing one particularly important growth in this cluster, namely, *Dementia* (from 0.51% to 1.1%).

Finally, the relative decline of the topic *Omission* is particularly surprising. All top‐10 papers either discuss the doctrine of double effect (DDE) or the distinction between killing and letting die (KLD), which were traditionally treated as crucial for many discussions about practical bioethics dilemmas, and most have been written by well‐known philosophers such as Philippa Foot, Frances M. Kamm, and Warren Quinn. Given the involvement of such prominent philosophers, one might expect sustained influence; yet, it seems that these classic themes are losing their relative popularity in bioethics.

#### 4.4.2 Peaks

The diachronic analysis of topic prominence allowed us to identify the most pronounced topic peaks, understood as the highest sudden increases of topic prominence (in a given 5‐year period, as compared to the previous two periods). From the top‐5 peaks (*Nudge* [2011–2015], *Embryos: research* [2001–2005], *Pharma ethics* [2001–2005], *Death: euthanasia* [1991–1995], and *Participation* [2001–2005]), we have already mentioned our interpretation of the first and fifth topics.

Both peaks of the topic *Embryos: research* (2001–2005 and 1991–1995) may be straightforwardly interpreted as reactions to scientific discoveries and breakthroughs. The most visible peak (2001–2005) represents a clear reaction to the discovery in 1997 and 1998 of methods for deriving and culturing human embryonic stem cells indefinitely and methods for the cloning of adult mammals using nuclear replacement techniques. Many papers that are assigned the highest probability to this peak period^57^ cite the influential paper by Thomson and colleagues published in *Science*,^58^ while others also discuss the regulations or guidelines that followed these discoveries (e.g., British Human Fertilisation and Embryology Act of 1990 amended in 2001; the U.S. Human Cloning Prohibition Act of 2001; German Embryonic Stem Cell Act from 2002—“Stammzellgesetz—StZG”; guidelines for human embryonic stem cell research published by the National Academy of Sciences in 2005). The earlier peak of the same topic (1991–1995) is a reaction to some advances in reproductive technology, in particular, in vitro fertilization (1978) and then cryopreservation (1984), that have made early human embryo experimentation a possibility. Many papers characteristic for this peak discuss both the practical aspects of these discoveries and the philosophical issues related to them (e.g., the problem of potentiality or identity of early embryos); half of them discuss or mention Warnock's Report of the Committee of Inquiry into Human Fertilisation and Embryology (1984) or some other contemporary legal (e.g., Australian Infertility Act 1984, the Report of the German Enquete Commission) or religious documents (the Vatican's Instruction on Respect for Human Life in Its Origins and on the Dignity of Procreation).

Skimming through the top‐30 documents in *Pharma Ethics* 2001–2005 provides a much more diversified picture: Some articles refer to particular political events such as the Congressional investigation in 2004 about the FDA's failure to release a report about the safety of children taking prescription antidepressants or new legal regulations (e.g., Prescription Drug User Fee Act and FDA Modernization Act from 2004 or Pharmaceutical Market Access Act from 2003). Some others discuss recently published important books on big pharma,^59^ but many others discuss more general issues such as the prices of drugs and basic research; ethical issues in industry gift‐giving; regulation of emergency contraceptives, overriding drug patents; internet pharmacies; the influence of pharmaceutical companies on medical education, and so forth.

In contrast, *Death: euthanasia* (1991–1995) can easily be explained by skimming through the top‐30 documents. First, 11 out of 30 papers engage with the Remmelink Committee report in September 1991 about practicing euthanasia in the Netherlands. Second, 12 out of 30 papers discuss contemporary attempts in the United States to legalize euthanasia (e.g., referenda in California 1988, Washington 1991, Oregon 1994), court judgments, and the 1993 Dr. Kevorkian case. This is a clear case when the increase of academic interest in a specific theme has a clear social origin.

### 4.5 Limitations

Our approach is mostly data‐driven and automatic, but it also includes “manual interventions” at several important junctures. Primarily, this concerns identifying the corpus and then assigning the number of topics and other parameters of the model, labeling topics, and, finally, clustering them. In particular, there are other possible approaches to constructing the corpus that could result in a different general picture of the field. Instead of delineating the most important bioethics journals, as we did, one could choose to collect the most important articles, defining “importance” as, for example, the most cited articles published in a larger set of journals. Therefore, the picture resulting from our modeling is not self‐evident and should not be treated as a ready‐made object created independently of any human intervention but rather as a useful tool or a piece of evidence that may help researchers in their own interpretive engagement with the original text materials.^60^

We assume that the observed distribution of topics and diachronic trends mirror the changing patterns in research interests, which in turn are reactions to scientific discoveries and breakthroughs, political events and legal decisions, important publications, and broader social trends and transformations. For this reason, in many cases, it is much easier to interpret the growth of interest in a particular theme than a decline. On a more concrete level, two significant increases in the prominence of topic *Embryos: research*, first in the late 1980s and the second in the early 2000s, or the very recent prominence of the topic *Germline* can be interpreted as reactions to actual scientific/technological breakthroughs. Some other trends clearly mirror societal or legal changes (e.g., the explosion of topic *Death: euthanasia* in the early 1990s), whereas still others reflect the changing landscape of social science and the humanities (e.g., the increased prominence of topic *Nudge* in the early 2010s).

However, interpreting the trends is not always an easy task. A topic model does not allow us to uncover the motives that made a given author use terms characteristic for one of our topics, and not the other, in a given text. Let us take the theme of religion as a concrete example: It appears that an average article in bioethics and philosophy of medicine is almost as likely to discuss religion today as it was half a century ago (the prominence of the topic *Religion* only dropped from 0.98% to 0.75%). However, is this theme still discussed in the same way? Or is it that, earlier, religious traditions used to be a source of inspiration for bioethical reflection, while today they are just another object of study? A topic model (or, at least, *this* topic model) does not offer a conclusive answer (as texts arguing in favor of the continuing importance of religion might be associated with the same topic as those pointing to the irrelevance of religion).

Even when one observes a clear trend of relative decline, it is not always evident how one should interpret it (e.g., does the relative decline of prominence of the END OF LIFE topics indicate a general decline in the interest in such themes?). Given that the whole corpus is expanding massively in time, a relative decline in prominence (at least) in many cases still means growth in absolute terms. Therefore, it is not clear whether (relative) decline actually signals a decrease in interest. It is also worth mentioning that some trends may merely result from the choices or preferences of editors, although the fact that we took seven journals into account should offset the editorial preferences of a single editor or an editorial board.

We limited our study to papers published in English *and* in the last 50 years. The reason behind the first limitation is a simple result of the fact that a text‐mining technique of the sort used here would not operate well on a multilingual corpus. We had to choose one language, and English has dominated the field for longer than the 50 years analyzed. Second, we chose the period not because we think that bioethics and philosophy of medicine started in 1971, but because it was only then that specialist journals started to emerge, marking the consolidation of the research community. None of these should be treated as suggesting that bioethics and philosophy of medicine consist solely of texts written in this language and in this period, nor as an endorsement of the state of affairs in which texts written in English dominate the debate in bioethics and philosophy of medicine. Philosophy of medicine may be traced back to Hippocratic tradition, and systematic humanistic reflection on ethical issues in medicine developed in the 19th century.^61^ Although the bulk of the discipline is in English and was published very recently, it would also be interesting to examine the state of bioethics and philosophy of medicine in other languages too (e.g., French, German, Polish, Spanish) and in other periods.

## 5 FINAL REMARKS

The topic model described in this article, at the most basic level, is a testament to the heterogeneity of bioethics and philosophy of medicine. The model consists of 100 topics, out of which 91 are interpreted as representing clearly distinct substantive areas of interest in the field. Even when we tried to reduce the dimensionality of this structure, it was only with eight different communities based on intertopic correlations that we found a satisfactory clustering. It is virtually impossible that any approach based on close reading would have resulted in such a fine‐grained, yet firmly rooted map of this diverse field.

The heterogeneity of this area is also reflected in the diverse set of diachronic trends that we have identified. First, there are themes that seem to follow a steady, monotonic trend throughout the analyzed period: some have been gradually going out of fashion (in particular, *History*, *Confidentiality*, *Science: philosophy*), whereas others have emerged as new and important areas of research (in particular, *Enhancement*, *Public health emergencies*, or *Circumcision*). This type of dynamic is arguably shared with most other academic disciplines: Scholarly interests typically resist rapid changes. However, what is more characteristic of the analyzed field is the second type of observed changes: sudden, rapid peaks in interest in some themes (in particular, *Nudge* [2011–2015], *Embryos: research* [2001–2005], *Pharma ethics* [2001–2005]). We interpret this second pattern as reflecting the aspiration of bioethics to react quickly to changing circumstances: on the one hand, to keep pace with most recent scientific and technological breakthroughs, while commenting on urgent social, political, and legal challenges on the other. Finally, despite the inherently dynamic nature of bioethics and philosophy of medicine, we still observed themes that perhaps surprisingly have not experienced any noticeable decline (or rise) in interest (in particular, *Brain*, *Rescue*, *Law: international*, *Phenomenology*).

Although this paper focused on partitioning the field of bioethics and philosophy of medicine into topics and describing diachronic trends, the model can also be used for further analyses. For instance, it can be used to characterize journals in terms of topic mixtures or look for correlations between topics and the demographic characteristics of authors.^62^ Furthermore, the model's output can also be used as a didactic tool. The graphs presented in this paper can serve as useful visual entry points for introductory discussions of bioethics and philosophy of medicine, starting from the very high‐level structure of the field in terms of topic clusters and then zooming into more fine‐grained topic distribution and diachronic trends. By providing extensive online supplements, we encourage readers not only to engage in their own interpretations of the present corpus but also to utilize the model in a variety of ways, from more focused historical analyses to teaching. We also hope that this study will motivate further corpus‐based research in philosophy in general and in bioethics and philosophy of medicine in particular, reaching into larger and more diverse—in terms of types of texts, historical periods, and languages—corpora.

## CONFLICT OF INTEREST

The authors declare no conflict of interest.

## Footnotes

^1^
Jonsen, A. R. (2003). *The birth of bioethics*. Oxford University Press.

^2^
Veatch, R. M. (2006). How philosophy of medicine has changed medical ethics. *The Journal of Medicine and Philosophy*, *31*(6), 585–600.

^3^
Jonsen, op. cit. note 1.

^4^
See important meta‐bioethical discussions in the 100th anniversary issue of *AJOB* published in 2013 (Vol. 13, no. 1); special issue “The Future of Bioethics” published by the journal *Bioethics* in 2010 (Vol. 24, no. 5); or an anniversary issue of *Journal of Medicine and Philosophy* published in 2006 (Vol. 31, no. 6).

^5^
Dawson, A. (2010). The future of bioethics: Three dogmas and a cup of hemlock. *Bioethics*, *24*(5), 218–225.

^6^
For a discussion of such criticisms: Pääkkönen, J., & Ylikoski, P. (2021). Humanistic interpretation and machine learning. *Synthese*, *199*(1), 1461–1497.

^7^
Moretti, F. (2013). *Distant reading*. Verso Books.

^8^
Blei, D. M., & Lafferty, J. D. (2009). Topic models. In A. N. Srivastava & M. Saha (Eds.), *Text mining*. Classification, Clustering, and Applications (pp. 101–124). Chapman and Hall/CRC; Griffiths, T. L., & Steyvers, M. (2004). Finding scientific topics. *Proceedings of the National Academy of Sciences*, *101*(Suppl. 1), 5228–5235; Blei, D. M., Ng, A. Y., & Jordan, M. I. (2003). Latent Dirichlet allocation. *The Journal of Machine Learning Research*, *3*, 993–1022.

^9^
Areas in which scholarly output has been analyzed using topic‐modeling techniques include, but are not limited to, anthropology (Marwick, B. (2013). Discovery of emergent issues and controversies in anthropology using text mining, topic modeling, and social network analysis of microblog content. In Y. Zhao & Y. Cen (Eds.), *Data mining applications with R* (pp. 63–93). Academic Press); cognitive science (Cohen Priva, U., & Austerweil, J. L. (2015). Analyzing the history of Cognition using Topic Models. *Cognition*, *135*, 4–9); computational linguistics (Hall, D., Jurafsky, D., & Manning, C. D. (2008). Studying the history of ideas using topic models. In M. Lapata & H. T Ng (Eds.), *Proceedings of the 2008 Conference on Empirical Methods in Natural Language Processing* (pp. 363–371). Omnipress Inc.); ecology (Andrew, N. R., Evans, M. J., Svejcar, L., Prendegast, K., Mata, L., Gibb, H., Stone, M. J.,  & Barton, P. S. (2022). What's hot and what's not—Identifying publication trends in insect ecology. *Austral Ecology*, *47*(1), 5–16); history (Mimno, D. (2012). Computational historiography: Data mining in a century of classics journals. *Journal on Computing and Cultural Heritage*, *5*(1), 1–19); history of science (Peirson, B. E., Bottino, E., Damerow, J. L., & Laubichler, M. D. (2017). Quantitative perspectives on fifty years of the Journal of the History of Biology. *Journal of the History of Biology*, *50*(4), 695–751); medicine (Porturas, T., & Taylor, R. A. (2021). Forty years of emergency medicine research: Uncovering research themes and trends through topic modeling. *The American Journal of Emergency Medicine*, *45*, 213–220); and, last but not least, philosophy (Malaterre, C., Lareau, F., Pulizzotto, D., & St‐Onge, J. (2021). Eight journals over eight decades: A computational topic‐modeling approach to contemporary philosophy of science. *Synthese*, *199*(1–2), 2883–2923; Weatherson, B. (2022). *A history of philosophy journals, I: Evidence from topic modeling, 1876–2013*. Michigan Publishing Services; Kinney, D. (2022). Diachronic trends in the topic distributions of formal epistemology abstracts. *Synthese*, *200*(1), 1–34).

^10^
Pääkkönen & Ylikoski, op. cit. note 6.

^11^
We use the word “topic” to refer to statistical patterns (revealed by our topic model) of co‐occurrence of words across documents in our corpus and the word “cluster” to refer to communities of topics determined by their tendency to be present together in the same documents in our corpus. We *italicize* the names of the topics and CAPITALIZE the names of the clusters. When we refer not to our corpus but to the discipline itself, we will instead use the terms “theme” and “area [of research].”

^12^
Malaterre, C., et al., op. cit. note 9; Weatherson, op. cit. note 9.

^13^
This approach implies that all and only texts that were published in the target journals were included in our analysis and each of them is given “equal weight.” The impact of any specific text (as measured, e.g., by citation statistics) plays no role in this study. Critically, some of the most influential texts in bioethics are not included in our corpus—simply because they were published in different outlets. However, even if some influential text is not included in our corpus, the ideas and themes it introduced should have later resonated in the target journals, so they are in no way lost.

^14^
Wencelius, J., Garine, E., & Raimond, C. (2017). *FLARES*. http://www.anthrocogs.com/shiny/flares/

^15^
Smith, J. J., & Borgatti, S. P. (1997). Salience counts‐and so does accuracy: Correcting and updating a measure for free‐list‐item salience. *Journal of Linguistic Anthropology*, *7*, 208–209. Sutrop, U. (2001). List task and a cognitive salience index. *Field Methods, 13*(3), 263–276.

^16^
For detailed characteristics of selected journals and the exact number of documents each of them contributed to the corpus, consult Online Supplement 1 (https://osf.io/5364e/).

^17^
The data were highly saturated, indicating a high level of cultural consensus. All the journals from the final top‐7 were already mentioned in the lists provided by the first six experts (who, in combination, mentioned 68% of all the journals in the final list). It took 13 experts (<50%) to mention >90% of all the journals in the full list.

^18^
More detailed lists of inclusion/exclusion criteria for each journal can be found in Online Supplement 2 (https://osf.io/ey6sn/).

^19^
Lopez, P. (2009). GROBID: Combining automatic bibliographic data recognition and term extraction for scholarship publications. In M. Agosti, J. Borbiha, S. Kapidakis, C. Papatheodorou, & G. Tsakonas (Eds.), *International Conference on Theory and Practice of Digital Libraries* (pp. 473–474). Springer.

^20^

*Stopwords* are words that are filtered out from a text before conducting a computational analysis on it. Most of the time, stopwords are words that are so common in the natural language that their presence in a given text does not provide any useful document‐specific information. An English‐language stopword list typically contains words such as *the*, *you*, *do*, and so forth.

^21^
Bird, S. (2006). NLTK: The natural language toolkit. In J. Curran (Ed.), *Proceedings of the COLING/ACL 2006 Interactive Presentation Sessions* (pp. 69–72). Association for Computational Linguistics.

^22^
Rehurek, R., & Sojka, P. (2010). Software framework for topic modelling with large corpora. In R. Witte, H. Cunningham, J. Patrick, E. Beisswanger, E. Buyko, U. Hahn, K. Verspoor, & A. R. Coden (Eds.), *Proceedings of the LREC 2010 Workshop on New Challenges for NLP Frameworks (pp. 51‐55)*. LREC.

^23^
Mikolov, T., Sutskever, I., Chen, K., Corrado, G. S., & Dean, J. (2013). Distributed representations of words and phrases and their compositionality. In *Advances in neural information processing systems* (pp. 3111–3119). Curran Associates Inc.

^24^

https://spacy.io/api/annotation

^25^
Lemmatization means replacing different inflected variants of a given word with the basic (dictionary) form of a given word (so that, e.g., *got* and *gets* are replaced with *get*).

^26^
Honnibal, M., & Montani, I. (2017). spaCy 2: Natural language understanding with Bloom embeddings, convolutional neural networks and incremental parsing. *7*(1), 411–420, To Appear.

^27^
Blei, D. M., et al., op. cit. note 8.

^28^

https://pypi.org/project/lda/

^29^
For nontechnical introductions to LDA algorithms, consult Mohr, J. W., & Bogdanov, P. (2013). Introduction—Topic models: What they are and why they matter. *Poetics*, *41*(6), 545–569; DiMaggio, P., Nag, M., & Blei, D. (2013). Exploiting affinities between topic modeling and the sociological perspective on culture: Application to newspaper coverage of US government arts funding. *Poetics*, *41*(6), 570–606.

^30^
Intuitively, a higher *alpha* results in documents assigning a nonnegligible probability to fewer topics, and a higher *beta* results in topics assigning nonnegligible probability to fewer terms.

^31^
The list of 10 terms most strongly associated with each topic can be found in Appendix A, while the list of 10 documents in which each topic is expressed most strongly can be found in Online Supplement 3 (https://bioethics.incet.uj.edu.pl/top10-documents-per-topic). See also the interactive LDAvis visualization of the 100 topics: https://bioethics.incet.uj.edu.pl/visualisation (Sievert, C., & Shirley, K. (2014). LDAvis: A method for visualizing and interpreting topics. In *Proceedings of the Workshop on Interactive Language Learning, Visualization, and Interfaces* (pp. 63–70)).

^32^
Another way of analyzing the links between terms and topics is to identify topics with which a given term is most strongly associated. In our context, this can be done with the following R Shiny app: https://bioethics.incet.uj.edu.pl/lexicon

^33^
The topic distribution for each document in the corpus can be examined with the following R Shiny app: https://bioethics.incet.uj.edu.pl/documents

^34^
The presence of uninterpretable jargon topics can be seen as evidence that the *K* value selected by the modeler is too large. In our context, however, jargon topics would not disappear even for the lowest tested number of topics (*K* = 30). Furthermore, the proportion of jargon topics in our preferred model (3 topics out of 100, with the joint prominence of 10.5%) is moderate and appears more than acceptable.

^35^
Cohen Priva & Austerweil, op. cit. note 9.

^36^
Although the distinction between jargon and framing topics is not yet a standard one in the literature, we find it useful. Jargon topics, at best, represent some particularities of style in which a text is written, which is not something relevant in a study like this one. Framing topics, on the other hand, represent distinct ways (in the sense of distinct genres or used methods) in which more contentful topics can be “framed” in a given document. For these reasons, we exclude jargon topics from all the analyses to follow but include framing topics whenever we think it could result in some relevant observations.

^37^
Throughout this paper, we refer to topics using the short labels. Those, however, can be occasionally misleading. For this reason, we encourage readers interested in specific topics to always consult the long label and the list of most likely terms in Appendix A.

^38^
Malaterre, C., et al., op. cit. note 9.

^39^
Such a correlation measures the degree to which given two topics are likely to be associated with the same documents. In other words, topics that are correlated have been often expressed in the same articles.

^40^
We set this threshold around the minimal value that allowed connecting all the topics to the main graph. Setting it at a higher value would allow us to retain only the most notable correlations at the expense of leaving many topics disconnected from the graph. Setting it at a lower value would introduce many small and uninformative correlations and make the graph less readable.

^41^
Bastian, M., Heymann, S., & Jacomy, M. (2009). Gephi: An open source software for exploring and manipulating networks. In *Third International AAAI Conference on Weblogs and Social Media* (pp. 361‐362); Blondel, V. D., Guillaume, J.‐L., Lambiotte, R., & Lefebvre, E. (2008). Fast unfolding of communities in large networks. *Journal of Statistical Mechanics: Theory and Experiment*, *2008*(10), P10008.

^42^
By overall prominence, we mean the arithmetic mean of probabilities assigned to a given topic by all the documents, see Appendix A, see also Online Supplement 4 (https://osf.io/pxjqh) for the table of topics ordered by prominence.

^43^
See Online Supplement 5 (https://osf.io/eqjsm).

^44^
For the full table, see Online Supplement 6 (https://osf.io/9pu5h).

^45^
The threshold was based on our judgment on what would be the best point separating shorter texts from standard research articles. The threshold of 2300 words allowed us, for example, to exclude from the abridged corpus 98% of documents labeled as “Open Peer Commentaries,” while retaining 90% of documents labeled as “Original Paper.” The abridged corpus contains 52% of documents from the full corpus and 80% of the overall word count.

^46^
For the list of recent changes for all topics, see Online Supplement 7 (https://osf.io/jx9dq).

^47^
DiMaggio P., et al., op. cit. note 29

^48^
Steinbock, B. (2011). Fetal sentience and women's rights. *Hastings Center Report*, *41*(6), c3.

^49^
Strong, C. (2008). A critique of “the best secular argument against abortion”. *Journal of Medical Ethics*, *34*(10), 727–731.

^50^
It is worth mentioning that the overall cluster structure does not obviously map on either the applied/theoretical distinction or the bioethics/philosophy of medicine distinction.

^51^
The interpretation of that cluster as characterized by a sense of novelty is further corroborated by the observation that the lists of the 20 most likely terms for topics from EMERGING TOPICS are on average “younger” than for topics from any other cluster (where a term's “age” is determined by the year in which it first appeared in our corpus).

^52^
However, the winners in terms of absolute growth are different, and all top three winners in this regard belong to the cluster PATIENTS AND RESEARCH PARTICIPANTS: *Participation* (which grew 1.64 percentage points, from 0.19% to 1.83%), *Risk and precaution* (1.4, from 0.59% to 1.99%), and *International research* (1.16, from 0.31% to 1.55%), with the topic *Enhancement* in the 10th place (0.94). At the same time, in the case of the biggest losers in absolute terms, they are almost identical to the relative losers: *Confidentiality* (−3.75), *History* (−2.8), and *Law: health* (−2.77), with the topic *Science: philosophy* in the 4th place (−2.48).

^53^
Cwik, B. (2020). Revising, correcting, and transferring genes. *The American Journal of Bioethics*, *20*(8), 7–18.

^54^
Benatar, M., & Benatar, D. (2003). Between prophylaxis and child abuse: the ethics of neonatal male circumcision. *American Journal of Bioethics*, *3*(2), 35–48.

^55^
Thaler, R. H., & Sunstein, C. R. (2008). *Nudge: Improving decisions about health, wealth, and happiness*. Yale University Press.

^56^
Blumenthal‐Barby, J. S. (2011). On the concept and measure of voluntariness: insights from behavioral economics and cognitive science. *The American Journal of Bioethics*, *11*(8), 25–26; Nelson, R. M., Beauchamp, T., Miller, V. A., Reynolds, W., Ittenbach, R. F., & Luce, M. F. (2011). The concept of voluntary consent. *The American Journal of Bioethics*, *11*(8), 6–16.

^57^
For the list of top‐30 documents for each identified peak and recent trend, see Online Supplement 9 (https://bioethics.incet.uj.edu.pl/top-documents-per-peak).

^58^
Thomson, J. A., Itskovitz‐Eldor, J., Shapiro, S. S., Waknitz, M. A., Swiergiel, J. J., Marshall, V. S., & Jones, J. M. (1998). Embryonic stem cell lines derived from human blastocysts. *Science*, *282*(5391), 1145–1147.

^59^
Such as Angell M. (2004). *The truth about the drug companies: How they deceive us and what to do about it*. Random.

^60^
See discussion about topic realism and instrumentalism in Pääkkönen & Ylikoski, op. cit. note 6.

^61^
Percival T. (1803), *Medical ethics; or, a code of institutes and precepts, adapted to the professional conduct of physicians and surgeons*. J. Johnson.

^62^
For an application of the present topic modeling study to evaluate the role of philosophy in bioethics see: Bystranowski, P., Dranseika, V., & Żuradzki, T. (2022). The disconnection that wasn't: Philosophy in modern bioethics from a quantitative perspective. *The American Journal of Bioethics*, Retrieved from http://philsci-archive.pitt.edu/id/eprint/21150.

## APPENDIX A TOPICS PER CLUSTER

### A.1

**Table:**

Topic Id	Short label	Long label	Top‐10 terms	Corpus prominence (%)
**BEGINNING OF LIFE**				
**11**	**Abortion: regulatory**	**Abortion: regulatory issues**	abortion fetus woman pregnancy fetal mother birth child pregnant prenatal	0.83
**28**	**Embryos: identity**	**Metaphysics of beginning of life**	organism identity capacity body personal soul biological entity brain twin	0.61
**30**	**Reproductive autonomy**	**Womens**’ **reproductive autonomy**	woman pregnancy birth pregnant mother reproductive child man feminist sterilization	0.66
**34**	**Neonatal**	**Neonatal intensive care**	infant parent baby child newborn birth bear death die neonate	0.66
**40**	**Embryos: research**	**Stem cells and embryo research**	embryo cell stem_cell status development embryonic_stem create egg tissue early	0.91
**73**	**Abortion: philosophy**	**Abortion: philosophical issues**	kill status future abortion fetus wrong personhood morally property being	0.70
**100**	**IVF and surrogacy**	**IVF and surrogacy**	child reproductive woman couple donor ivf sperm parent egg mother	0.89
**END OF LIFE**				
**5**	**Death: life support**	**Withholding/Withdrawal of life support**	withdraw die death sustain withdrawal wish family refuse continue prolong	0.97
**8**	**Dementia**	**Decision making in dementia**	dementia capacity preference surrogate advance_directive competent competence incompetent wish ad	0.99
**27**	**Death: concept**	**Concept of death**	death brain dead function criterion definition organ whole loss die	1.03
**48**	**Law: health**	**Health law**	law court legal rule judge protect protection supreme_court statute criminal	1.85
**63**	**War and prisons**	**(Medical) ethics in war and prisons**	prisoner military prison war victim torture crime criminal violence punishment	0.74
**69**	**Death: euthanasia**	**Euthanasia and physician‐assisted suicide**	euthanasia death die suicide physician request assisted_suicide assist suffer pas	1.03
**76**	**Omissions**	**Doctrine of double effect and act/omission distinction**	action kill cause morally death agent intend intention distinction wrong	0.78
**81**	**Hazards**	**Risks and occupational hazards**	injury worker compensation employer employee damage injure job cause exposure	0.34
**85**	**Sedation**	**Pain and palliative sedation**	pain suffer suffering palliative sedation death symptom relief relieve distress	0.44
**INSTITUTIONS**				
**19**	**Insurance**	**Health insurance**	cost service insurance pay government plan program economic private coverage	1.86
**21**	**Public health emergencies**	**Public health emergencies**	healthcare covid pandemic triage emergency worker resource crisis provider disaster	0.64
**23**	**Nursing**	**Nursing ethics**	nurse nursing home caregiver professional nursing_home family burden physical hospital	0.36
**41**	**Resuscitation**	**Resuscitation**	family cpr futility resuscitation intervention physician surrogate futile death intensive	1.04
**52**	**Vaccination**	**Vaccination and communicable diseases**	vaccine disease aid infection vaccination transmission virus infect antibiotic population	0.85
**53**	**Justice**	**Justice and equality**	justice equality opportunity inequality capability basic fair theory equal distribution	1.00
**54**	**Ageing**	**Ageing**	age old elderly young death extend extension adult population future	0.42
**55**	**Resources**	**Resource allocation**	resource priority cost allocation save criterion ration chance equal qaly	1.11
**84**	**Hospital**	**Patient ‐ hospital relations**	hospital staff team mr service member family home institution facility	1.23
**PATIENTS AND RESEARCH PARTICIPANTS**				
**13**	**RCT**	**RCT**	trial intervention standard design evidence therapy control drug equipoise rct	1.37
**15**	**Biobanking**	**Biobanking**	datum sample consent tissue biobank material researcher data database project	0.61
**20**	**Family decisions**	**Family‐based decision making**	family member chinese china confucian japanese cultural culture western japan	0.63
**37**	**Addiction**	**Substance dependence**	drug medication addiction addict prescription prescribe control opioid substance user	0.58
**39**	**Nudge**	**Nudging in public health**	choice option influence nudge preference choose bias nudging rational default	0.53
**42**	**Consent**	**Consent**	consent inform informed procedure obtain disclosure requirement standard disclose autonomy	1.10
**44**	**Screening**	**Cancer screening and overdiagnosis**	disease cancer blood therapy diagnosis cure chronic early illness cause	1.00
**45**	**Global**	**Global bioethics**	country global international world population poor government economic nation resource	0.95
**61**	**Participation**	**Research participation**	participant researcher participation trial participate investigator consent conduct phase recruitment	1.31
**62**	**Circumcision**	**Circumcision and genital mutilation**	circumcision procedure perform dental cultural male_circumcision circumcise infant male amputation	0.31
**64**	**Monetary incentives**	**Monetary incentives**	market payment pay sell exploitation money kidney incentive compensation coercion	0.68
**70**	**Surgery**	**Ethical issues in surgery**	surgery procedure surgeon surgical device heart operation perform dbs implant	0.60
**79**	**Organ donation**	**Organ donation**	organ donor transplant donation transplantation donate recipient kidney organ_donation family	1.22
**80**	**International research**	**International research ethics**	community researcher member share project local global resource engagement population	0.85
**89**	**Responsibility**	**Responsibility in public health**	responsibility responsible choice smoke behaviour food control behavior obesity alcohol	0.58
**91**	**Committees**	**Research ethics committees**	committee review irb member regulation report guideline protocol institution commission	1.78
**92**	**Autonomy**	**Autonomy**	autonomy self choice autonomous freedom action paternalism capacity agent free	1.37
**93**	**Risk and precaution**	**Harm, risks, and precautionary principle**	harm justify intervention standard harmful serious prevent threshold protect minimal	1.43
**95**	**Children: decisions**	**Children in decision making**	child parent parental adult adolescent family pediatric minor age young	1.20
**97**	**Vulnerability**	**Vulnerability in research**	vulnerability vulnerable experiment protection experimentation consent experimental protect researcher therapeutic	0.39
**99**	**Law: international**	**International and comparative law**	law country legal european article committee guideline german international declaration	1.22
**PHILOSOPHY OF MEDICINE**				
**22**	**Wellbeing**	**Health and wellbeing**	desire rational theory goal ability objective belief account quality happiness	0.62
**56**	**Statistics**	**Statistical methods and measurement**	outcome measure evidence probability quality datum analysis assessment method factor	1.22
**65**	**Psychiatry**	**Psychiatry and mental disorders**	mental disorder psychiatric illness psychiatry psychiatrist depression symptom diagnosis schizophrenia	0.82
**74**	**Science: philosophy**	**Medicine and general philosophy of science**	science theory scientific model causal knowledge explanation cause philosophy mechanism	1.20
**82**	**Disease: concept**	**Concepts of disease and health**	disease concept function definition normal goal illness account define theory	0.70
**83**	**Diagnosis**	**Medical diagnosis and epistemology**	knowledge diagnosis diagnostic expert epistemic evidence judgment reasoning clinician uncertainty	0.61
**94**	**Brain**	**Consciousness and brain**	brain consciousness memory function cognitive mind behavior activity response neuroscience	0.78
**PHYSICIAN AND RESEARCHER**				
**6**	**Physician**	**Physician's role**	physician relationship conflict professional doctor treat obligation judgment primary profession	1.78
**16**	**Trust**	**Trust‐based relationships**	relationship trust gift contract relational power party share model relation	0.51
**17**	**Consultation**	**Ethics consultation**	consultation consultant ethicist service committee expertise consult cec member team	0.90
**18**	**Codes**	**Professional ethics and ethics codes**	professional profession code client standard member practitioner obligation service physician	0.73
**32**	**Rescue**	**Duty to rescue**	duty obligation rescue responsibility special morally beneficence harm action treat	0.84
**35**	**Medical error**	**Medical error and professionalism**	resident error rural professionalism report professional hour physician colleague safety	0.31
**38**	**Pharma ethics**	**Pharma ethics**	drug company product industry fda market consumer regulation access regulatory	1.05
**57**	**Confidentiality**	**Medical confidentiality**	doctor confidentiality professional dr practitioner relationship profession hospital duty tell	1.35
**75**	**Publication**	**Publication ethics**	journal author article publish publication report paper scientific academic review	1.01
**78**	**Placebo**	**Placebo**	placebo deception lie truth belief deceive tell false cam homeopathy	0.41
**86**	**Ethics education**	**Ethics education**	student education school teach program learn training teaching skill professional	1.25
**96**	**Conscientious objection**	**Conscientious objection**	conscience integrity objection conscientious_objection professional service abortion refuse provider belief	0.51
**98**	**Privacy**	**Privacy: digital**	privacy user datum technology record medium access online personal internet	0.68
**EMERGING TOPICS**				
**1**	**Germline**	**Germline modification and gene therapy**	genetic gene therapy clone cloning disease intervention technology engineering germline	0.57
**14**	**Genetic testing**	**Genetic testing**	test genetic testing screen disease screening family disclosure diagnosis finding	1.12
**26**	**Animals**	**Animals and chimeras**	animal experiment specie being status capacity primate chimera mouse chimpanzee	0.51
**29**	**Sexuality**	**Sexuality**	sexual sex partner man love relationship homosexual homosexuality marriage sexuality	0.29
**31**	**Disability**	**Disability**	disability disabled impairment deaf autism disable autistic language model hear	0.44
**33**	**Enhancement**	**(Moral) enhancement**	enhancement enhance technology intervention cognitive capacity trait morally improve bioenhancement	0.62
**46**	**Ecology**	**Conservation and ecology**	natural nature specie biological environment environmental organism evolutionary evolution biology	0.47
**47**	**Patenting**	**Patenting and new technologies**	technology science scientific scientist patent development technological application knowledge innovation	0.87
**51**	**Sports**	**Fairness in sports**	solidarity sport athlete player performance team game club play doping	0.28
**68**	**Offspring**	**Obligations to offspring**	child parent genetic choice pgd reproductive future harm selection bear	0.78
**72**	**Gender**	**Sex and gender**	gender sex female male woman man identity girl sex_selection ashley	0.45
**88**	**Genetics**	**Genetics: concepts and research**	genetic gene genomic genome dna disease trait sequence environmental factor	0.66
**THEORETICAL BIOETHICS**				
**4**	**Phenomenology**	**Phenomenological bioethics**	world illness self heidegger meaning body object understanding possibility existential	0.96
**7**	**Biopolitics**	**Biopolitics**	power political discourse cultural culture self modern institution technology community	1.47
**10**	**Principlism**	**Principlism debate**	theory morality norm judgment rule common normative method practical justification	1.47
**25**	**Deliberation**	**Health policy and democratic deliberation**	policy political debate consensus government deliberation citizen position democratic member	1.07
**36**	**Narratives**	**Narrative ethics**	narrative story metaphor tell identity language text image write illness	0.60
**43**	**Dignity**	**Dignity**	dignity kant being kantian treat concept worth capacity rational self	0.40
**50**	**Religion**	**(Abrahamic) Religious bioethics**	religious religion god belief tradition christian secular islamic muslim catholic	0.86
**58**	**Body: ownership**	**Body ownership**	body bodily property object physical ownership aesthetic material self embody	0.41
**59**	**Metabioethics**	**Metabioethics**	bioethic bioethicist bioethical field discipline science academic philosophy debate perspective	1.25
**67**	**Empathy**	**Empathy and moral distress**	emotion distress empathy emotional feel hope feeling response attitude self	0.69
**77**	**History**	**Historical topics in medicine and philosophy of medicine**	man nature world modern philosophy mind soul history word god	1.07
**87**	**Virtue ethics**	**Virtue ethics**	virtue action character pellegrino aristotle practical wisdom virtuous agent knowledge	0.48
**90**	**Race**	**Race**	race white black racial community population cultural disparity racism minority	0.72
**FRAMING TOPICS**				
**2**	**Concepts**	**Definitions of concepts**	concept definition kind define notion nature criterion account distinction refer	2.55
**12**	**Qualitative**	**Qualitative empirical bioethics**	interview participant professional empirical perspective analysis feel datum qualitative et_al	1.29
**24**	**Quantitative**	**Quantitative empirical bioethics**	respondent survey table response report questionnaire difference attitude datum participant	1.30
**49**	**Moral philosophy**	**Moral philosophy discourse**	objection morally relevant non kind justify account thing position conclusion	3.10
**60**	**Clinical stories**	**Clinical stories**	get tell feel day mother thing die look talk family	2.17
**71**	**Reviews**	**Book reviews**	book chapter author philosophy philosophical essay reader philosopher write analysis	1.63
**JARGON**				
**3**	**Trash1**	**Trash. Kind, thing, simply**	kind thing simply get indeed far try always choice yet	4.61
**9**	**TrashBE**	**Trash (British English)**	programme healthcare recognise service non evidence behaviour professional paper nhs	1.93
**66**	**Trash3**	**Trash. Clinician, challenge, goal**	clinician challenge goal model framework identify author colleague intervention policy	3.99

